# Long-wavelength *Mesh&Collect* native SAD phasing from microcrystals

**DOI:** 10.1107/S2059798319002031

**Published:** 2019-02-11

**Authors:** Michele Cianci, Max Nanao, Thomas R. Schneider

**Affiliations:** a Department of Agricultural, Food and Environmental Sciences, Università Politecnica delle Marche, Via Brecce Bianche, 60131 Ancona, Italy; b EMBL Grenoble, 71 Avenue des Martyrs, CS 90181, 38042 Grenoble, France; c EMBL, Notkestrasse 85, 22607 Hamburg, Germany

**Keywords:** microcrystals, long wavelength, mesh, genetic algorithm, phasing

## Abstract

Harnessing the anomalous signal from macromolecular crystals with volumes of less than 10 000 µm^3^ for native phasing requires careful experimental planning. The type of anomalous scatterers that are naturally present in the sample, such as sulfur, phosphorus and calcium, will dictate the beam energy required and determine the level of radiation sensitivity, while the crystal size will dictate the beam size and the sample-mounting technique, in turn indicating the specifications of a suitable beamline. On the EMBL beamline P13 at PETRA III, *Mesh&Collect* data collection from concanavalin A microcrystals with linear dimensions of ∼20 µm or less using an accordingly sized microbeam at a wavelength of 1.892 Å (6.551 keV, close to the Mn edge at 6.549 keV) increases the expected Bijvoet ratio to 2.1% from an expected 0.7% at 12.6 keV (Se *K* edge), thus allowing experimental phase determination using the anomalous signal from naturally present Mn^2+^ and Ca^2+^ ions. Dozens of crystals were harvested and flash-cryocooled in micro-meshes, rapidly screened for diffraction (less than a minute per loop) and then used for serial *Mesh&Collect* collection of about 298 partial data sets (10° of crystal rotation per sample). The partial data sets were integrated and scaled. A genetic algorithm for combining partial data sets was used to select those to be merged into a single data set. This final data set showed high completeness, high multiplicity and sufficient anomalous signal to locate the anomalous scatterers, and provided phasing information which allowed complete auto-tracing of the polypeptide chain. To allow the complete experiment to run in less than 2 h, a practically acceptable time frame, the diffractometer and detector had to run together with limited manual intervention. The combination of several cutting-edge components allowed accurate anomalous signal to be measured from small crystals.

## Abstract 2

A long-wavelength mesh data collection using a size-tailored microbeam from concanavalin A microcrystals with linear dimensions of less than 20 µm allowed experimental phase determination using the anomalous signal from naturally occurring Mn^2+^ and Ca^2+^ ions.

## 1. Introduction   

*De novo* determination of macromolecular structures requires the accurate measurement of structure factors and retrieval of experimental phases from the crystals of the given specimen. When a model with significant structure similarities is available, phases can be retrieved using the molecular-replacement (MR) method. Otherwise, the phases must be determined experimentally. One experimental phasing method that is gaining popularity is the use of the anomalous signal from naturally occurring anomalous (native SAD) or from *ad hoc* incorporated anomalous scatterers (*International Tables for Crystallography*, 2012 ▸).

However, along with the growing popularity of anomalous scattering for phasing, X-ray radiation damage became a general concern for any data collection performed on an undulator beamline, resulting in systematic analyses of synchrotron data sets at room temperature or cryotemperatures, with samples showing the characteristic ‘fingerprints’ of radiation damage (Helliwell, 1988 ▸; Ravelli & McSweeney, 2000 ▸; Borek *et al.*, 2007 ▸). Radiation damage still represents a potential issue (Ravelli & Garman, 2006 ▸) for data collection, but may also provide an opportunity to collect additional phasing information (Ravelli *et al.*, 2003 ▸; Nanao *et al.*, 2005 ▸). Data collection from microcrystals of macromolecular compounds can make use of multi-crystal diffraction data collection (Smith *et al.*, 2012 ▸) to overcome radiation damage to the sample (Owen *et al.*, 2006 ▸).

The advantages of a multi-data-set data-collection strategy have been theoretically analysed by Liu *et al.* (2011 ▸), showing a reduction in the background noise of the diffraction data compared with the commonly used single-data-set strategy, with clear benefits for native and anomalous data collections. Liu *et al.* (2012 ▸) showed how multi-crystal data collection for a sulfur SAD experiment (reviewed in Rose *et al.*, 2015 ▸) enabled the solution of several crystal structures at medium to low resolution from crystals with linear dimensions of ∼200 µm.

Liu *et al.* (2011 ▸) estimated the wavelength for the optimum transmitted anomalous signal on the basis of sample size, X-ray absorption and incoherent scattering to be approximately 2.06 Å (6 keV), 2.47 Å (5 keV) and 3.1 Å (4 keV) for typical crystal sizes of 200, 100 and 50 µm, respectively. The use of a long wavelength increases the anomalous signal from several native atoms. For instance, data collection at λ = 2.69 Å (4.6 keV) from crystals of 50 µm in size allowed the determination of the crystal structure of Cdc23^Nterm^, a subunit of the multimeric anaphase-promoting complex (APC/C), at 3.1 Å resolution by sulfur SAD phasing. At this energy, Cdc23^Nterm^ had an expected Bijvoet ratio 〈|Δ*F*_anom_|〉/〈*F*〉 of 2.2% compared with 0.45% at λ = 1 Å (12.6 keV) (Cianci *et al.*, 2016 ▸). Increasing the expected Bijvoet ratio 〈|Δ*F*_anom_|〉/〈*F*〉 by choosing an appropriate wavelength decreases the requirement in *I*/σ(*I*) for successful phasing, as discussed by Cianci *et al.* (2016 ▸).

Microcrystallography has advanced by using the ability to accurately locate crystals embedded in opaque matrices by rastering a mounted sample using micrometre-sized X-ray beams to test each point of the sample for diffraction, as in the case of crystals embedded in lipidic cubic phase (Cherezov *et al.*, 2009 ▸; Warren *et al.*, 2013 ▸). Serial crystallography using synchrotron radiation has recently shown that complete data sets can be compiled from data collected from a cryocooled vitrified suspension of *in vivo*-grown micrometre-sized protein crystals (Gati *et al.*, 2014 ▸) by pumping a suspension of protein microcrystals at room temperature across the path of the X-ray beam in a glass capillary (Stellato *et al.*, 2014 ▸) or at room temperature in a slowly flowing free-standing high-viscosity microstream (Botha *et al.*, 2015 ▸).

In a further step towards the optimal collection of diffraction data in synchrotron serial crystallography, an automatic workflow has been developed in which many randomly oriented diffracting microcrystals are identified on a single cryocooled sample holder using a two-dimensional X-ray-based scan followed by the collection of partial data sets with online processing (Zander *et al.*, 2015 ▸). Using this protocol, crystals of *Bacillus thermoproteolyticus* thermolysin (rod-shaped crystals of between 40 × 40 × 150 and 40 × 40 × 300 µm in size) were phased using an X-ray beam of 10 µm at the Zn *K* absorption edge (1.282 Å, 9.761 keV) and crystals of the MAEL domain of *Bombyx mori* Maelstrom (20–50 µm in the largest dimension) were phased using an X-ray beam of 10 µm at the Se *K* absorption edge (0.979 Å, 12.6 keV). In the latter case, 45 partial data sets were merged for the final data set to enable structure solution.

As oscillation ranges are collected using this method, the partiality of the reflections can be determined and combined to obtain a complete and high-quality data set. One problem that remains, however, is dealing with the non-isomorphism between crystals, which is highly dependent on the system being studied, crystal nucleation, microenvironment growth conditions, crystal mounting and cryoprotection methods. For a set of *n* partial data sets the number of possible combinations is 2^*n*^ − 1, and an exhaustive search quickly becomes computationally demanding even with a small number of partial data sets. Genetic algorithms (GAs), which are well known global optimization methods that have already been applied to address diverse problems in macromolecular crystallo­graphy (Chang & Lewis, 1994 ▸; Kissinger *et al.*, 1999 ▸; Schneider, 2002 ▸; Uervirojnangkoorn *et al.*, 2013 ▸), have been proven to be a powerful tool to group partial data sets for merging into a high-quality data set (Zander *et al.*, 2016 ▸).

We show here that when long wavelengths (Djinovic Carugo *et al.*, 2005 ▸) are combined with the *Mesh&Collect* data-collection approach (Zander *et al.*, 2015 ▸) and when a genetic algorithm (GA) is used to compile a data set (Zander *et al.*, 2016 ▸), a native SAD (Rose *et al.*, 2015 ▸) experiment can yield a structure solution from microcrystals. Finally, optimization of the X-ray scanning routines and data-collection flows allowed thousands of microcrystals to be screened in just a few hours.

## 2. Materials and methods   

### 2.1. Crystallization and crystal mounting   

**Figure 1:**
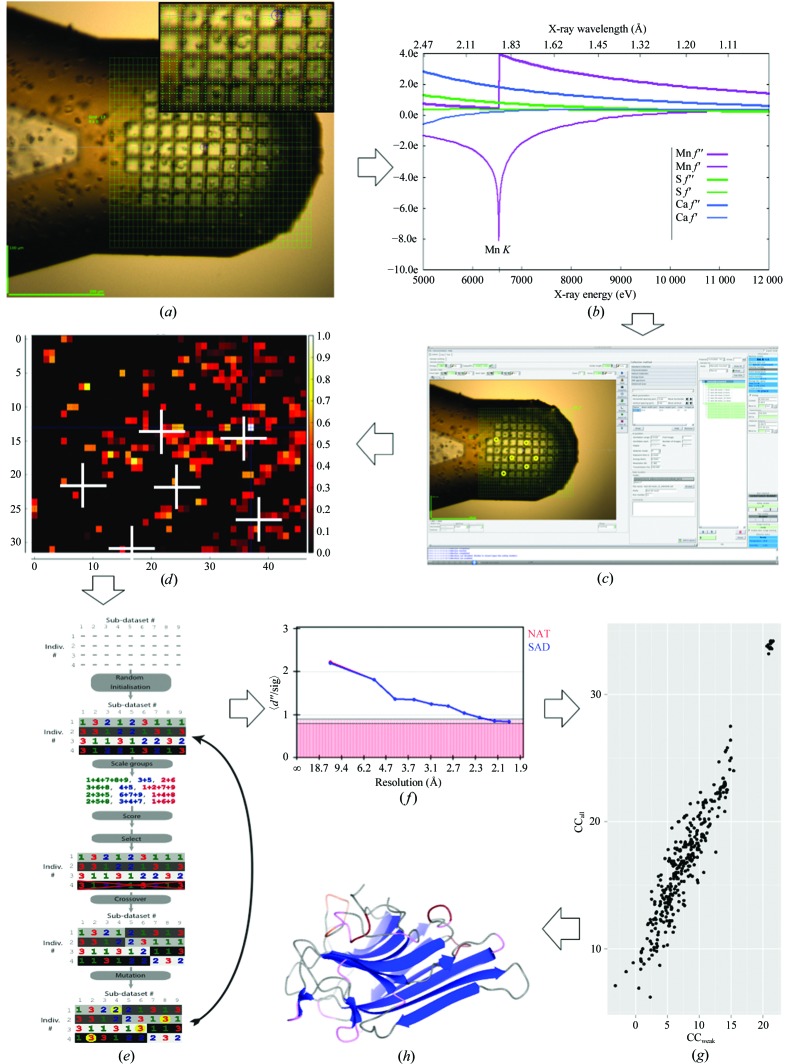
The workflow for long-wavelength *Mesh&Collect* native SAD phasing data collection from ConA microcrystals and structure solution. (*a*) ConA microcrystals scooped onto a mesh. The scan grid is drawn to indicate the region of interest with *MXCuBE*2. Grid squares are sized to 15 × 15 µm according to the beam cross-section selected. (*b*) The wavelength is selected to optimize the expected Bijvoet ratio for the protein. (*c*) A grid scan is performed on the sample; each grid point is scored for diffraction and the result is presented as a heat map within *MXCuBE*2. (*d*) Heat-map colours from dark red (low) to yellow (high) represent the diffraction intensity as a function of position within the region of interest; white crosses mark the positions (*x*, *y*) that have been selected and used for the collection of partial data sets. For *x* and *y*, the unit is the beam size. Positions for partial data collections and common data-collection parameters are selected for each data point and the data-collection queue is launched. (*e*) Partial data sets are automatically processed with *XDS* and selected with the GA (Zander *et al.*, 2016 ▸) to produce an optimized final data set for structure solution based on the optimization of *I*/σ(*I*), *R*_merge_ and CC_1/2_. (*f*) Plot of 〈Δ_ano_/σΔ_ano_〉 *versus* resolution for the GA-optimized final data set. (*g*) Scatter plot of CC_weak_ versus CC_all_ from *SHELXD*. (*h*) Refined model. Location of anomalous scatterers, phasing and refinement follow.

The lectin concanavalin A (ConA; UniProt entry P02866) contains 237 amino-acid residues with two methionines and binds one Mn^2+^ cation and one Ca^2+^ cation (Deacon *et al.*, 1997 ▸). Crystals of ConA (Fluka product No. 61760, lot No. 420479/1) were grown within one week by the hanging-drop method from drops consisting of equal amounts of protein solution (1 µl; 10 mg ml^−1^ in water) and reservoir solution [1 µl; 34%(*v*/*v*) PEG 1500] buffered with 5 m*M* HEPES pH 6.0. Crystallization conditions that yielded showers of crystals with a maximum linear dimension of ∼20 µm or less were obtained starting from the conditions previously reported by Mueller-Dieckmann *et al.* (2005 ▸). ConA crystals were scooped directly from the crystallization drop onto a 25 µm MiTeGen mesh and were flash-cooled to 100 K in a gaseous stream of nitrogen (Fig. 1 ▸ *a*). The crystals belonged to space group *I*222, with unit-cell parameters *a* = 61.6, *b* = 85.6, *c* = 88.8 Å.

### 2.2. Data collection and processing   

Diffraction data were collected at 100 K using synchrotron radiation on the EMBL beamline P13 at the PETRA III storage ring, c/o DESY, Hamburg, Germany (Cianci *et al.*, 2017 ▸; Table 1 ▸). P13 is equipped with an Arinax MD2 diffractometer (Perrakis, Cipriani *et al.*, 1999 ▸; Bowler *et al.*, 2010 ▸) featuring a 240 MHz PMAC CPU for fast grid scanning and a high-resolution CCD camera (GigE, 1/2′′, 1360 × 1024 pixels, colour) for the MD2 on-axis video microscope (Cipriani *et al.*, 2007 ▸). The standard detector on P13 is a PILATUS 6M-F hybrid pixel-array detector (Dectris, Baden, Switzerland) with 450 µm sensor thickness and custom calibration tables for low energies (Marchal *et al.*, 2011 ▸; Marchal & Wagner, 2011 ▸), which was operated in shutterless data-collection mode at the maximum frame rate of 25 Hz. An Amptek XR-100SDD fluorescence detector (Amptek, Bedford, Massachusetts, USA) was used to perform an X-ray fluorescence scan on ConA crystals mounted on a test mesh and determined the Mn edge peak position. The peak position and the inflection points were determined at 6.549 and 6.545 keV, respectively. The Si(111) double-crystal monochromator was subsequently set to a wavelength of 1.892 Å (6.551 keV, close to the Mn edge at 6.549 keV) with an unattenuated X-ray photon flux of 1.36 × 10^11^ photons s^−1^ throughout the 15 µm collimator aperture, which was selected to match the average crystal size, and was used for both the mesh scans and the partial data-set collections.

Choosing a wavelength of 1.892 Å (Fig. 1 ▸ *b*) increased the expected Bijvoet ratio for ConA to 2.1% from an expected value of 0.7% at 0.976 Å (12.6 keV, Se *K* edge), as calculated using the equation proposed for mixed-element anomalous scatterers by Olczak *et al.* (2003 ▸) via the *ASSC* (*Anomalous Scattering Signal Calculator*) web server (http://assc.p.lodz.pl/) and the *ProtParam* tool on the ExPASy web server (http://web.expasy.org/protparam/; Gasteiger *et al.*, 2005 ▸).

The protocol applied for data collection has been described as *Mesh&Collect* (Zander *et al.*, 2015 ▸). In our experiment, after being mounted on the goniometer head using the MARVIN sample changer available at the beamline (Cianci *et al.*, 2017 ▸), each mesh was carefully aligned perpendicularly to the incoming photon beam. Using *MXCuBE* (Gabadinho *et al.*, 2010 ▸), a grid was depicted over the MiTeGen mesh, with a periodicity of 15 µm selected according to the beam cross-section (Fig. 1 ▸ *c*). The horizontal and vertical movements of the mesh with respect to the beam was via the two sampX and sampY motors, while no rotation of the goniometer axis was performed. Each grid point was then scored for diffraction using *Dozor* (Zander *et al.*, 2015 ▸; Popov & Bourenkov, 2016 ▸) followed by the generation of a diffraction heat map (Bowler *et al.*, 2010 ▸). The top ten maxima in the diffraction heat map were selected for the collection of partial data sets (±5°; Fig. 1 ▸ *d*). All data were automatically integrated on-the-fly using *XDS* (Kabsch, 2010 ▸). When necessary, data sets were automatically re-indexed for consistency across all partial data sets using the REFERENCE_DATA_SET keyword in *XDS*. The choice of the partial data sets to be merged into a high-quality data set (Tables 2 ▸ and 3 ▸) was performed by the genetic algorithm described in Zander *et al.* (2016 ▸) (Fig. 1 ▸ *e*) to produce a final data set with good anomalous signal (Fig. 1 ▸ *f*). SAD phasing was performed with *SHELXC*, *SHELXD* and *SHELXE* (Schneider & Sheldrick, 2002 ▸; Sheldrick, 2004 ▸, 2015 ▸; Fig. 1 ▸ *g*). Ten rounds of autobuilding using *ARP*/*wARP* with sequence docking (Perrakis, Morris *et al.*, 1999 ▸) and manual refinement with *REFMAC*5 (Murshudov *et al.*, 2011 ▸; Winn *et al.*, 2011 ▸) and *Coot* (Emsley *et al.*, 2010 ▸) gave *R*-factor and *R*_free_ values of 0.15 and 0.184, respectively (Table 4 ▸, Figs. 1 ▸ *h* and 2 ▸). Phase errors against the reference structures were computed in *SHELXE*.

## 3. Results and discussion   

### 3.1. Collection of partial data sets   

Native SAD experiments are considered to be challenging and critically dependent on the collection of accurate data (Rose *et al.*, 2015 ▸). Thus, we were interested in whether it was possible to collect small wedges of data from micrometre-sized crystals at a long wavelength in order to merge them and harness the anomalous signal from native anomalous scatterers to produce interpretable phases. The data-collection strategy was to enhance the expected Bijvoet ratio 〈Δ*F*_anom_〉/〈*F*〉 by choosing a wavelength of 1.892 Å at the Mn edge to increase the anomalous signal of Mn^2+^ and Ca^2+^ ions in the softer X-ray regime (Rose *et al.*, 2015 ▸). Data were collected to the maximum resolution possible of 1.929 Å owing to the geometry of the camera and the wavelength.

The crystals were scooped directly from the drop using a grid as a support. The distribution of the crystals with a random orientation in many crystal arrays allows data collection over the complete reciprocal space, without the need for multi-axis goniometry, as shown by the high multiplicity and the high completeness seen in the data-collection statistics (Table 3 ▸).

For the grid scans and partial data-set collection, an un­attenuated X-ray beam and the maximum detector frame rate of 25 Hz were used to make use of the full potential of beamlines at third-generation X-ray sources such as PETRA III at DESY.

A typical grid scan of 50 × 30 points covering a region of interest of 750 × 450 µm, with a 40 ms exposure time per point in shutterless operation, could be performed in about a minute (see Supplementary Movie) thanks to the highly optimized goniometer hardware and control software. The complete heat map was available shortly after each grid scan and, typically, the top ten points were used for data collection. The exposure time for each grid point was 40 ms, with a photon flux of 1.36 × 10^11^ photons s^−1^ at the Mn edge. This was equivalent to an X-ray dose of 0.04 MGy, which is equivalent to ∼0.2% of the Henderson limit, thus preserving most of the of the lifetime of the crystals for subsequent data collection.

For the ConA crystals, with a size of ∼20 µm or less, we used a ±5° wedge (Table 1 ▸). Limiting the rotation range to a 10° wedge for each data-collection position reduces the requirements in terms of the sphere of confusion of the diffraction spindle, since for small overall rotations the crystal will not move out of the photon beam cross-section, so that a two-dimensional centring is sufficient, as previously discussed by Zander *et al.* (2015 ▸). For each partial data-set collection, the overall exposure time was limited to 4 s with an unattenuated beam. According to calculations with *RADDOSE* (Paithankar *et al.*, 2009 ▸), this was equivalent to an overall X-ray dose of about 4 MGy, or one fifth of the Henderson–Garman limit of 20 MGy (Henderson, 1990 ▸; Owen *et al.*, 2006 ▸).

With a mesh scan taking 60 s and with an overall time per grid point of 4 s, data collection from a single grid, including the travelling time between data-collection positions, took less than 3 min when including overhead time owing to the sample changer. The data collection (30 grid scans for 298 partial data sets) was completed within 2 h. The autoprocessing routines could index and integrate 180 partial data sets out of the 298 that were collected. As the purpose of this experiment was to limit the manual intervention to the bare minimum at any step, the unindexed data sets were not considered further. Visual inspection of images that failed indexing revealed signs of multiple lattices. This problem can be ascribed to too dense a crystal distribution on the meshes and/or to cluster-grown crystals. The occurrence of such situations can in principle be minimized by dilution of the crystal droplets prior to scooping and by optimization of the crystal-growth conditions. Another possibility is to optimize the beam size to each single crystal before data collection, so as to avoid exposing multiple lattices to the X-ray beam.

### 3.2. Assembly of a full data set   

The genetic algorithm (Zander *et al.*, 2016 ▸; Foos *et al.*, 2018 ▸) has been developed for the production of high-quality data sets by combining partial data sets based on *I*/σ(*I*), CC_ano_ and CC_overall_ as quality indicators.

In brief, as described in Section 2.7 of Zander *et al.* (2016 ▸), GAs apply concepts of biological natural selection to maximize or minimize a target function. The problem being optimized is encoded into one or more chromosomes, which are contained in a population of randomly initialized individuals. Diversity is introduced into the population via random mutation and crossover events. A chromosome therefore simply describes how all of the sub-data sets should be divided into groups, with one sub-data set belonging only to one group. The GA algorithm then proceeds as follows: a population of individuals, each containing a single chromosome, is first randomly initialized (Fig. 1 ▸ *e*) and then undergoes cycles of GA optimization by repeated selection, crossover between individuals, mutations and evaluations of fitness. The evaluation of individuals is performed by first scaling together all of the sub-data sets in the chromosome with the same group number with *XSCALE* (Kabsch, 2010 ▸). After *XSCALE* has been executed, data-quality statistics are parsed from the XSCALE.Lp file and a fitness is calculated as a combination of the inner-shell *R*_meas_ value, the inner-shell 〈*I*/σ(*I*)〉, the outer-shell CC_1/2_ and the anomalous signal CC_ano_ according to the chosen weighting scheme to produce a single score for each group in the individual (Zander *et al.*, 2016 ▸). It was discovered that convergence of the GA required a relatively large number of cycles and a larger population size than the default value. Therefore, a systematic gridding of parameter weights was deemed to be impractical. Instead, several values for each weight were chosen, and the combination that yielded the best merging statistics was chosen. CC_anom overall_ was the primary sort criteria, followed by CC_1/2 overall_, then 〈*I*/σ(*I*)〉_overall_ and finally *R*_meas inner_. The weights that were used for these statistics were 5, 300, 1000 and 100, respectively (Table 2 ▸). The target function is the sum of each statistical value multiplied by the respective weight. The *R*_meas_ term, however, is 100 − *R*_meas overall_ × *R*_weight_ (for more details of the weighting and target functions, see Foos *et al.*, 2019 ▸). Finally, no cutoffs were required or applied for the minimum correlation or minimum *R*_meas_ used to sort the data sets.

A complete run of the GA with the parameters reported in Table 2 ▸ took 17 h of wall-clock time on a ten-core machine. The aggregated data set, obtained from the merging of 116 partial data-set wedges, resulted in high overall completeness and decent data quality, which allowed the location of the anomalous scatterers, successful phasing and structure refinement using standard methodology. The data-quality indicators show that it is possible to merge more than a hundred partial data sets collected using softer X-rays with excellent results and harness the anomalous signal (Fig. 1 ▸ *f*). Merging GA-selected data yielded an excellent CC_1/2_ as well as 〈*I*/σ(*I*)〉 (Tables 2 ▸ and 3 ▸). The 〈*I*/σ(*I*)〉 value is within the expected range of 18–55 estimated as necessary for successful SAD phasing (Olczak & Cianci, 2018 ▸). *R*_meas inner_ was 9.4% and *R*_meas overall_ was 14.8% (Table 2 ▸). The mean anomalous differences divided by their standard deviation (SigAno in *XSCALE*) indicated the presence of anomalous signal up to 2.0 Å resolution (Fig. 1 ▸ *f*), which was therefore chosen as the resolution cutoff for substructure determination.

### 3.3. Structure solution   

*SHELXD* correctly determined the Ca^2+^ and Mn^2+^ ions and the S atoms at the Met42 and Met129 sites, producing CC_all_, CC_weak_ and PATFOM values of 34.2, 21.7 and 55.8, respectively. The anomalous difference Fourier map peak heights were 46σ for Mn^2+^, 27σ for Ca^2+^, 7.5σ for the Met129 SD atom and 6.3σ for the Met42 SD atom, as assigned using *ANODE* (Thorn & Sheldrick, 2011 ▸). Interpretable phases were obtained with *SHELXE* after two rounds of solvent flattening with one round of automatic chain tracing. A best weighted mean phase error (wMPE) of 34° and a CC for the partial model of 22% were obtained in *SHELXE*, with 119 amino acids automatically built. A useful indicator of the validity of the anomalous atom sites for phasing is *R*_Cullis_, which is defined as 〈|observed − calculated anomalous difference|〉/〈|observed anomalous difference|〉. For anomalous data an *R*_Cullis_ of less than 1 is considered to provide significant phasing information. The *R*_Cullis_ calculated using *MLPHARE* (Winn *et al.*, 2011 ▸) starting from the Ca^2+^ and Mn^2+^ ions and the two S atoms from the methionine sites was 0.82. The *R*_Cullis_ values calculated starting from the Ca^2+^ ion or the Mn^2+^ ion alone were 0.90 and 0.89, respectively, indicating that in the case of proteins of analogous size but lacking cysteines or methionines a Ca^2+^ ion or the Mn^2+^ ion alone could provide significant phasing information.

Finally, no evidence of radiation damage was present in the electron-density maps for the crystal structure of ConA.

## 4. Conclusions and perspectives   

The linear dimensions of macromolecular crystals that are considered to be usable for X-ray data collection are continally becoming smaller and smaller, potentially reaching the point where crystals will be too small for optical centring and for individual data collection, but large enough to be detectable with low-dose X-ray centering and partial data-set collection. The availability of a high-intensity and well collimated beam, as on the P13 beamline (Cianci *et al.*, 2017 ▸), permits tailoring of the beam size to the sample size thanks to variable apertures, thus minimizing the scattering background.

Here, we have shown that the automated *Mesh&Collect* data-collection scheme as implemented at EMBL Hamburg is capable of automatically (i) locating sub-20 µm crystals in a matrix mounted on a standard mounting mesh, (ii) ranking these crystals by diffraction strength and (iii) collecting small rotation data sets at long wavelength from the highest ranking crystals under X-ray dose conditions.

Furthermore, the GA algorithm can effectively generate a highly complete data set from hundreds of naturally multi-oriented samples collected at long wavelength, thus permitting the compilation of a highly complete data set with high multiplicity, and thus preserving a weak anomalous signal and enabling structure solution by standard SAD phasing procedures.

As it stands, data collection at P13 is considerably faster than the GA data selection and processing, so the GA cannot effectively be deployed as a diagnostic tool for stopping data collection at the exact structure-solution time point, since by then is it most likely that more data than are needed have already been collected. Further improvements in these directions could come from parallel data processing, but new detector technology (for example, the Dectris EIGER 4M pixel detector capable of collecting diffraction patterns at a frame rate of 750 Hz) will inevitably increase the demand in CPU power.

Further enhancements in data quality could be achieved with optimized mounts to reduce background noise (Romoli *et al.*, 2014 ▸) and/or by tailoring the beam size and shape individually to each crystal to improve the signal to noise (Sanishvili *et al.*, 2008 ▸; Fischetti *et al.*, 2009 ▸). In summary, *Mesh&Collect* data collection using softer X-rays extends SAD phasing from naturally occurring anomalous scatterers to micrometre-sized crystals.

## Supplementary Material

PDB reference: concanavalin A, 6h2m

Supplementary Movie.. DOI: 10.1107/S2059798319002031/ap5033sup1.mp4

## Figures and Tables

**Figure 2:**
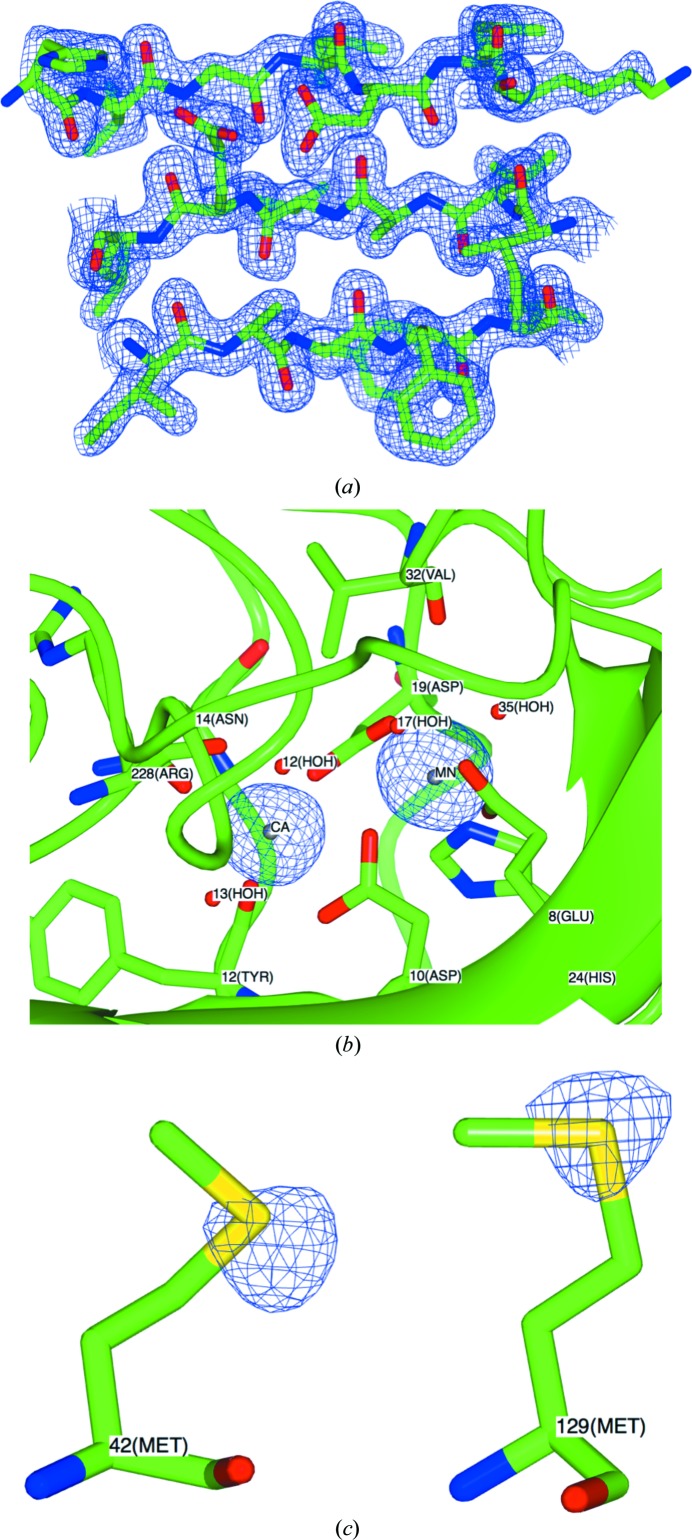
(*a*) Details of the final 2*mF*_obs_ − *DF*_calc_, α_calc_ Fourier electron-density map (contoured at 1.5 r.m.s.), with the refined structure shown in stick representation and standard atom colour-coding. (*b*, *c*) Anomalous difference (Δ*F*_anom_, α_calc_) Fourier electron-density map, contoured at 4.0 r.m.s., for (*b*) the Ca^2+^ and Mn^2+^ ions and (*c*) the S atoms (yellow) at the Met42 and Met129 sites. Graphics were produced using *CCP*4*mg* (McNicholas *et al.*, 2011 ▸).

**Table 1:** Data-collection parameters for mesh partial data sets of concanavalin A

Wavelength (Å)	1.892
Beam diameter (µm)	15
Linear crystal dimension range (µm)	5–20
Photon flux (photons s^−1^)	1.36 × 10^11^
Exposure per image (ms)	40
No. of images	100
Total exposure time per crystal (s)	4
Dose per partial data set (average diffraction-weighted dose) (MGy)	4.0
Oscillation range (°)	0.1
Total angular range per partial data set (°)	10

**Table 2:** Genetic algorithm (GA) and summary of the final merging statistics for concanavalin A In a GA, each iteration, or GA generation, results in a series of possible individuals for best approximating a function, and the GA population refers to the complete set or pool of these generated individuals after a given iteration. Each target also has a user-specified weight associated with it. All targets are then summed to produce a single fitness score for each group in the individual. For additional details, refer to Zander *et al.* (2016 ▸).

No. of partial data sets collected	298
No. of partial data sets integrated	180
No. of partial data sets selected	116
GA population size (individuals)	50
GA generations	400
GA *R* target weight	100
GA *I* target weight	1000
GA CC_1/2_ weight	300
GA groups	3
Resolution range	42.83–1.929 (1.998–1.929)
Total No. of reflections†	9145, 21675, 619871
No. of unique reflections†	379, 2389, 34104
Completeness† (%)	99.2, 94.7, 99.6
Multiplicity†	24.1, 9.1, 18.2
*R* value† (%)	9.20, 43.2, 14.4
*R*_meas_ † (%)	9.4, 45.8, 14.8
〈*I*/σ(*I*)〉†	50.41, 4.93, 18.89
SigAno†	2.287, 0784, 1.061
CC_1/2_ †	99.8, 94.1, 99.9

† The values reported are for the inner shell, for the outer shell and overall, respectively.

**Table 3:** Complete data statistics for the final data set obtained by scaling and merging the GA-selected subsets Correlation that is significant at the 0.1% level is marked by an asterisk.

	No. of reflections				*R* factor (%)						
Resolution (Å)	Observed	Unique	Multiplicity (%)	Completeness (%)	Observed	Expected	*I*/σ(*I*)	*R*_meas_ (%)	CC_1/2_	Anomalous correlation	SigAno
8.63	9145	379	24.1	99.20	9.20	9.30	50.41	9.40	99.8*	78*	2.287
6.10	14970	713	21.0	100.00	9.20	9.90	40.53	9.40	99.9*	68*	1.784
4.98	20198	901	22.4	100.00	9.20	10.00	41.49	9.40	99.9*	54*	1.444
4.31	24602	1062	23.2	100.00	9.30	9.90	43.48	9.50	99.9*	50*	1.424
3.86	26461	1217	21.7	100.00	10.40	10.40	38.27	10.70	99.9*	51*	1.467
3.52	28336	1376	20.6	100.00	10.90	10.80	35.42	11.20	99.8*	42*	1.363
3.26	31643	1434	22.1	100.00	12.10	11.90	32.93	12.40	99.8*	38*	1.324
3.05	35540	1603	22.2	100.00	12.80	12.70	30.16	13.10	99.8*	40*	1.307
2.88	36141	1661	21.8	100.00	14.50	14.70	25.04	14.90	99.7*	36*	1.200
2.73	34731	1781	19.5	100.00	17.10	17.20	20.68	17.60	99.7*	28*	1.121
2.60	38745	1857	20.9	100.00	19.50	19.80	18.91	20.00	99.5*	32*	1.118
2.49	40295	1959	20.6	100.00	21.90	22.40	16.46	22.40	99.4*	16*	0.976
2.39	40278	2020	19.9	100.00	24.30	25.00	14.61	25.00	99.3*	20*	0.987
2.31	36102	2089	17.3	100.00	25.70	26.50	12.82	26.50	99.2*	19*	0.951
2.23	38690	2199	17.6	100.00	28.20	29.10	12.10	29.00	99.1*	7	0.848
2.16	38479	2235	17.2	100.00	32.00	33.40	10.56	33.00	98.8*	12*	0.884
2.09	35830	2366	15.1	100.00	34.90	36.20	9.04	36.10	98.6*	9	0.867
2.03	35816	2395	15.0	100.00	38.60	40.90	8.01	40.00	98.2*	9	0.844
1.98	32194	2468	13.0	100.00	38.00	41.40	6.95	39.60	98.6*	8	0.837
1.93	21675	2389	9.1	94.70	43.20	47.90	4.93	45.80	94.1*	0	0.784
Total	619871	34104	18.2	99.60	14.40	14.80	18.89	14.80	99.9*	24*	1.061

**Table 4:** Data, phasing and structure-refinement statistics of concanavalin A

Data collection	
Space group	*I*222
Unit-cell parameters	61.6, 85.6, 88.8
Resolution† (Å)	42.83–1.929 (1.998–1.929)
Phasing statistics	
Best *SHELXE* CC of partial model	37.96
Average fragment size	21
Best MPE against partially refined model	64
Refinement statistics	
*R* factor (%)	0.15
*R*_free_ ‡ (%)	0.184
Cruickshank’s DPI for coordinate error§ based on *R* factor (Å)	0.14
Wilson plot *B* factor (Å^2^)	17.2
Average all-atom *B* factor (Å^2^)	19.8
R.m.s.d., bonds (Å)	0.004
R.m.s.d., angles (Å)	1.07
Total No. of non-H atoms	2076
Total No. of water molecules	260
Solvent content (%)	46.4
Matthews coefficient (Å^3^ Da^−1^)	2.3
Ramachandran plot¶	
Favoured region (%)	97.45
Allowed region (%)	2.13
Outliers (%)	0.43

† Values in parentheses are for the highest resolution bin.

‡ *R*_free_ is calculated using 5% of the total reflections that were randomly selected and excluded from refinement.

§ DPI = [*N*_atoms_/(*N*_refl_ − *N*_params_)]^1/2^ *RD*_max_ *C* ^−1/3^, where *N*_atoms_ is the number of atoms included in the refinement, *N*_refl_ is the number of reflections included in the refinement, *R* is the *R* factor, *D*_max_ is the maximum resolution of the reflections included in the refinement, *C* is the completeness of the observed data and, for isotropic refinement, *N*_params_ ≃ 4*N*_atoms_ (Cruickshank, 1999 ▸).

¶ Calculated with *PHENIX* (Adams *et al.*, 2010 ▸).
